# *Copaifera langsdorffii* oleoresin and its isolated compounds: antibacterial effect and antiproliferative activity in cancer cell lines

**DOI:** 10.1186/s12906-015-0961-4

**Published:** 2015-12-21

**Authors:** Fariza Abrão, Luciana Delfino de Araújo Costa, Jacqueline Morais Alves, Juliana Marques Senedese, Pâmela Tinti de Castro, Sérgio Ricardo Ambrósio, Rodrigo Cássio Sola Veneziani, Jairo Kenupp Bastos, Denise Crispim Tavares, Carlos Henrique G. Martins

**Affiliations:** Laboratório de Pesquisa em Microbiologia Aplicada, Universidade de Franca, Franca, São Paulo Brazil; Laboratório de Mutagênese, Universidade de Franca, Franca, São Paulo Brazil; Grupo de Pesquisa em Produtos Naturais, Universidade de Franca, Franca, São Paulo Brazil; Faculdade de Ciências Farmacêuticas de Ribeirão Preto, Universidade de São Paulo, Ribeirão Preto, São Paulo Brazil

**Keywords:** Diterpenes, Cytotoxicity, Multiresistant bacteria, Antibacterial activity, Copalic acid

## Abstract

**Background:**

Natural products display numerous therapeutic properties (e.g., antibacterial activity), providing the population with countless benefits. Therefore, the search for novel biologically active, naturally occurring compounds is extremely important. The present paper describes the antibacterial action of the *Copaifera langsdorffii* oleoresin and ten compounds isolated from this oleoresin against multiresistant bacteria; it also reports the antiproliferative activity of the *Copaifera langsdorffii* oleoresin and (-)-copalic acid.

**Methods:**

MICs and MBCs were used to determine the antibacterial activity. Time-kill curve assays provided the time that was necessary for the bacteria to die. The Minimum Inhbitory Concentration of Biofilm (CIMB_50_) of the compounds that displayed the best results was calculated. Cytotoxicity was measured by using the XTT assay.

**Results:**

The diterpene (-)-copalic acid was the most active antibacterial and afforded promising Minimum Inhibitory Concentration (MIC) values for most of the tested strains. Determination of the bactericidal kinetics against some bacteria revealed that the bactericidal effect emerged within six hours of incubation for *Streptococcus pneumoniae*. Concerning the antibiofilm action of this diterpene, its MICB_50_ was twofold larger than its CBM against *S. capitis* and *S. pneumoniae*. The XTT assay helped to evaluate the cytotoxic effect; results are expressed as IC_50_. The most pronounced antiproliferative effect arose in tumor cell lines treated with (-)-copalic acid; the lowest IC_50_ value was found for the human glioblastoma cell line.

**Conclusions:**

The diterpene (-)-copalic acid is a potential lead for the development of new selective antimicrobial agents to treat infections caused by Gram-positive multiresistant microorganisms, in both the sessile and planktonic mode. This diterpene is also a good candidate to develop anticancer drugs.

## Background

The discovery of antimicrobials has enabled physicians to treat diseases that used to have poor prognosis; however, resistant strains emerged shortly after microbicidal agents came into clinical use. The indiscriminate use of antibiotics has culminated in multiresistant bacterial strains [1–3], a situation that has prompted researchers to search for new bactericides in sources like medicinal plants [4, 5].

In nature, microorganisms generally fix onto surfaces; a matrix consisting of extracellular polymeric substances synthesized by the microbes themselves incorporates the microorganisms, creating a sessile population designated biofilm [6]. Microorganisms associated on a surface exhibit high growth rates and marked antimicrobial resistance [7]. Researchers have long attempted to develop new biologically active compounds against many multiresistant bacterial strains [8]; in this context, medicinal plants have arisen as a promising strategy—their antibacterial activity is easy to test via reliable and reproducible in vitro assays [2].

Cancer currently constitutes one of the leading causes of death worldwide; chemotherapy and/or radiotherapy are the main strategies available to treat this disease. The treatment is usually difficult, due to drug resistance, toxicity and low specificity [9]. Since ancient times, plants, particularly their secondary metabolites, have played an important part in cancer treatment and have led to the discovery of several effective anticancer agents that act directly or indirectly on the tumor [10].

The “Copaiba” oleoresin is extremely interesting from a chemical viewpoint: it contains a wide variety of secondary metabolites, mainly diterpenes and sesquiterpenes [11]. Although literature papers have reported that diterpenes and sesquiterpenes of the genus *Copaifera* display many biological activities, few publications on the antibacterial action of the species *Copaifera langsdorffii* exist [12–14]. The present work aimed to evaluate the antibacterial and cytotoxic activities of the *C. langsdorffii* oleoresin and its isolated compounds, to contribute to (1) the development of drugs that can prevent the bacterial growth through blockage of underlying mechanisms for multiresistant infections and (2) the prevention of cancer progression.

## Methods

### Plant material and compounds isolation

The authentic oleoresin from *Copaifera langsdorffii* was kindly provided by the Brazilian company Apis-Flora Comercial and Industrial. About 20.0 g of the oleoresin was fractionated by several chromatographic techniques, as described by Souza et al. [14]. These procedures furnished the compounds OC-1 (caryophylene oxide, 117.0 mg); OC-2 ((-) copalic acid, 450.0 mg); OC-3 (kaurenoic acid); OC-4 ((-)-acetoxycopalic acid, 230.0 mg); OC-5 ((-)-agathic acid, 150.0 mg); and OC-6 ((-)-hydroxycopalic acid, 130.0 mg). About 100.0 mg of the compounds OC-2, OC-4, OC-5 and OC-6 was treated with ethereal diazomethane. After addition of a small amount of acetic acid (to destroy the remaining diazomethane) and solvent removal, the derivatives OC-7, OC-8, OC-9 and OC-10 were obtained as described by Souza et al. [14]. The ^1^H and ^13^C-NMR spectral data indicated that the purity of each isolated compound ranged from 95 to 98 %.

### Antibacterial assay

#### Bacterial strains

To evaluate the antibacterial activity of the tested compounds, multiresistant clinical isolates and bacterial strains from the American Type Culture Collection (ATCC), namely *Staphylococcus epidermidis* (isolated from blood), *Staphylococcus capitis* (isolate from blood), *Staphylococcus haemolyticus* (isolate from blood), *Enterococcus faecalis* (isolated from urine), *Staphylococcus aureus* (isolated from surgical wound), *Staphylococcus aureus* (isolated from catheter), *Streptococcus pneumoniae* (isolated from blood), *Pseudomonas aeruginosa* (isolated from urine), *Acinetobacter calcoaceticus* (isolated from urine), *Escherichia coli* (isolated from urine), *Klebsiella pneumoniae* (isolated from urine), *Staphylococcus aureus* (WB81-USA 400), *Staphylococcus aureus* (W7749-USA 200), *Enterococcus faecium* (isolate from hospital outbreak), *Enterococcus faecium* (ATCC 19434), *Staphylococcus aureus* (ATCC 29213), *Staphylococcus capitis* (ATCC 27840), *Staphylococcus epidermidis* (ATCC 14990), *Staphylococcus haemolyticus* (ATCC 29970) and *Enterococcus faecalis* (ATCC 19433), were employed. The multiresistant clinical isolates were kindly supplied by Hospital das Clínicas de Ribeirão Preto (state of São Paulo, Brazil). The antibacterial activity of the isolated compounds was also tested against potentially pathogenic bacterial strains—*Kocuria rhizophila* (ATCC 9341), *Streptococcus pyogenes* (ATCC 19615), *Streptococcus pneumoniae* (ATCC 6305), *Enterococcus hirae* (ATCC 10541), *Staphylococcus aureus* (ATCC 9144), *Staphylococcus aureus* (ATCC 6538), *Bacillus subtillis* (ATCC 6051), *Bacillus cereus* (ATCC 14579), *Streptococcus dysgalactiae* (ATCC 9926), *Streptococcus agalactiae* (ATCC 27591), *Staphylococcus epidermidis* (ATCC 12228), *Enterobacter aerogenes* (ATCC 13048), *Pseudomonas aeruginosa* (ATCC 27853), *Escherichia coli* (ATCC 14948), *Proteus mirabilis* (ATCC 29906), *Morganella morganii* (ATCC 25829), *Citrobacter freundii* (ATCC 8090) and *Shigella flexneri* (ATCC 12022).

### Determination of the Minimum Inhibitory Concentration and Minimum Bactericidal Concentration

The Minimum Inhibitory Concentration (MIC) values were determined by the microdilution broth method in 96-well microplates, in triplicate [15]. The samples were dissolved in dimethylsulfoxide (DMSO, Sigma-Aldrich) at 1.0 mg mL^−1^, followed by dilution in Triptic Soy broth (Difco, Kansas City, MO, USA); concentrations ranging from 0.2 to 200.0 µg mL^−1^ were achieved. The final DMSO content was 5 % (v/v). The inoculum was adjusted for each organism, to yield a cell concentration of 5 × 10^5^ colony forming units (CFU) per mL, according to guidelines of the Clinical and Laboratory Standards Institute (CLSI). DMSO 5 % (v/v) was used as negative control; vancomycin and gentamicin were employed as positive control. One inoculated well was included, to control broth adequacy for bacterium growth. Another well containing broth free of antimicrobial agent and inoculum was included, to control medium sterility. The microplates (96 wells) were incubated at 37 °C for 24 h. After the incubation period, 30 μL of resazurin aqueous solution (0.02 %) was added to the microplates [16] for immediate observation of bacterial growth: the blue and red color indicated absence and presence of bacterial growth, respectively. The microplates were re-incubated for 30 min; then, they were analyzed in a descriptive way. To determine MBC, an aliquot of the inoculum was removed from each well before addition of resazurin and plated onto tryptic soy agar. Microorganism growth was detected after the incubation period and compared with the readings obtained in the microplates (MIC). This provided the concentration that was bactericidal—Minimum Bactericidal Concentration (MBC), defined as the lowest concentration of the compound that did not generate visible microbial growth in the medium. MCB was determined for the most active compound evaluated in this report.

#### Time-kill curves

Time-kill assays were performed in triplicate, based on D’arrigo et al. [17]. The average values were plotted in the graphs. The selected times for this evaluation were: 0, 30 min, 2, 4, 6, 12 and 24 h. Compound OC-2 was chosen for the time-kill curve assays because it was the most active. The concentration used during the test was based on the MBC value. The following microorganisms were evaluated: *S. aureus* (isolated from surgical wound), *S. capitis* (isolated from blood), *S. epidermidis* (isolated from blood), *S. haemolyticus* (isolated from blood), *E. faecalis* (isolated from urine), and *S. pneumoniae* (isolated from blood), because they displayed MBC lower than 100 µg mL^−1^.

#### Antibiofilm activity evaluation

The Minimum Inhibitory Concentration of Biofilm (MICB_50_) was determined on the basis of the minor concentration of the antibacterial agent that was able to inhibit 50 % or more of the biofilm. A microtiter plate assay based on CLSI [15] was used, with some modifications. Compound OC-2 was selected for antibiofilm activity evaluation; it was tested at concentrations ranging between 0.98 and 2000 µg mL^−1^. The bacterial strains were added at a concentration of 1 × 10^6^ CFU mL^−1^. Vancomycin was tested as negative control; bacterial strains in the absence of antibacterial agent were used as positive control. After a certain time of bacterial growth, biofilm production was quantified as described previously by Stepanović et al. [18], on the basis of the colorimetric measurement of crystal violet incorporated by sessile cells. The antibiofilm activity was also quantified by using the number of microorganisms (CFU mL^−1^).

### Cytotoxicity assay

#### Cell and culture conditions

Different cell lines were employed in this study: normal cell line, Chinese hamster lung fibroblasts (V79), and the tumor cell lines murine melanoma (B16F10), human breast adenocarcinoma (MCF-7), human cervical adenocarcinoma (HeLa), human hepatocellular liver carcinoma (HepG2), and human glioblastoma (MO59J, U343 and U251). The cell lines were maintained as monolayers in plastic culture flasks (25 cm^2^) in culture medium (HAM-F10 + DMEM, 1:1 or only DMEM, Sigma-Aldrich) supplemented with 10 % fetal bovine serum (Nutricell), antibiotics (0.01 mg mL^−1^ streptomycin and 0.005 mg mL^−1^ penicillin; Sigma-Aldrich), and 2.38 mg mL^−1^ Hepes (Sigma-Aldrich), at 37 °C, with 5 % CO_2_ or in a BOD-type chamber.

#### Antiproliferative activity

Cytotoxicity was measured by using the in vitro Toxicology Colorimetric Assay Kit (XTT; Roche Diagnostics) according to the manufacturer’s instructions. For these experiments, 1 × 10^4^ cells were plated onto 96-well microplates. Each well received 100 μL of HAM-F10/DMEM or DMEM medium containing *C. langsdorffii* oleoresin at concentrations ranging from 15 to 7630 µg mL^−1^ and OC-2 at concentrations ranging from 1.21 to 9830 μg mL^−1^. The negative (without treatment), solvent (Tween 80 0.25 %), and positive controls—doxorubicin (DXR, Zodiac), (S)-(-)-camptothecin (CPT, Sigma-Aldrich) and etoposide (VP16, Sigma-Aldrich)—were included. After incubation at 37 °C for 24 h, the medium was removed. The cells were washed twice with 100 μL of phosphate buffered saline (PBS) and exposed to 100 μL of HAM-F10 medium without phenol red. Then, 50 μL of XTT were added to each well. The microplates were covered and incubated at 37 °C, for 17 h. The absorbance of the samples was determined by using a multiplate reader (ELISA, Tecan – SW Magellan vs 5.03 STD 2PC) at a test wavelength of 492 nm and a reference wavelength of 690 nm [19]. The experiments were accomplished in triplicate. The antiproliferative activity was assessed with the aid of the parameter of 50 % inhibition of cell line growth (IC_50_) and selectivity index (SI, normal cells IC_50_/tumor cells IC_50_). The experiments were conducted in triplicate.

## Results and Discussion

**Fig. 1:**
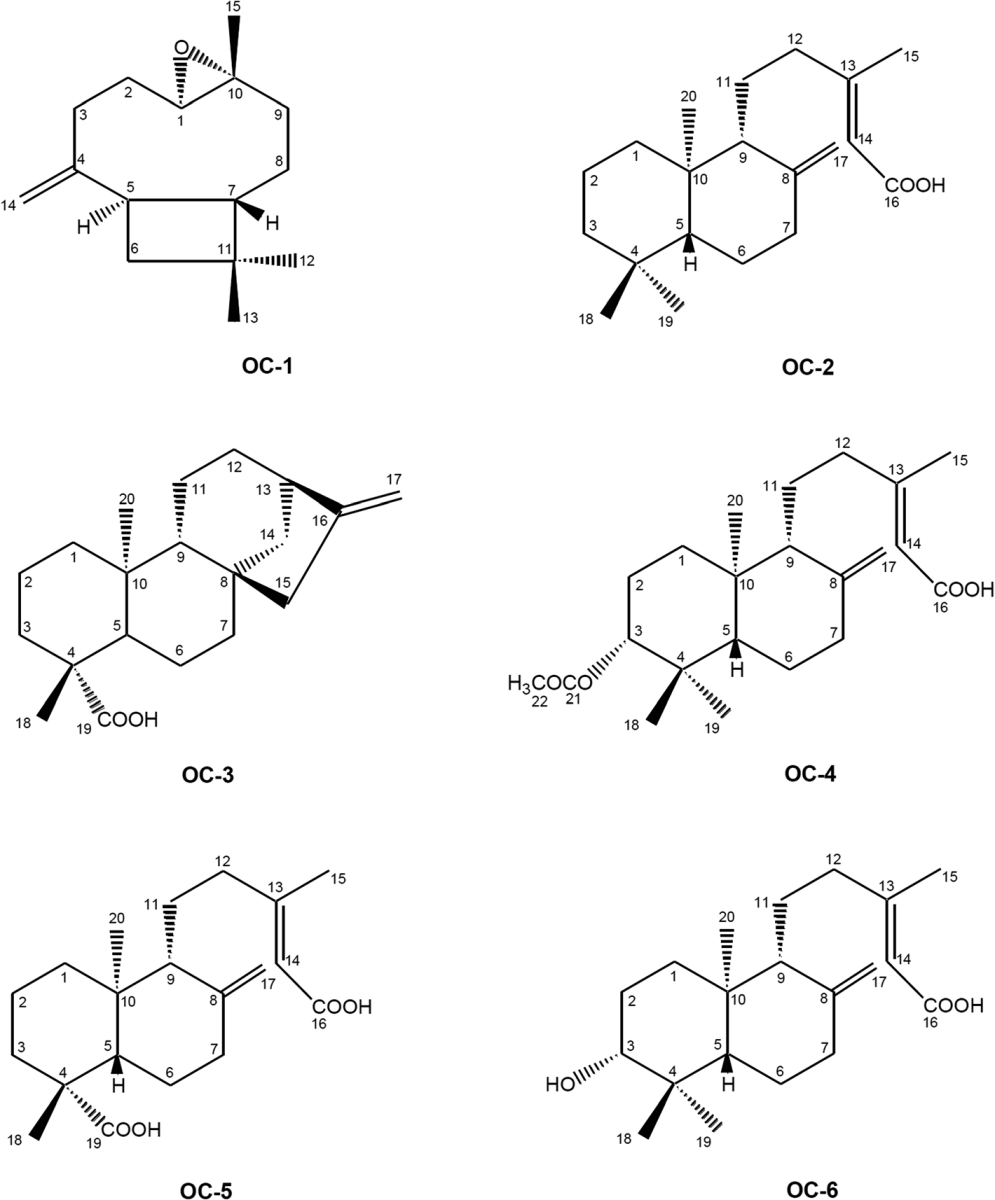
Chemical structures of the evaluated compounds

**Fig. 2:**
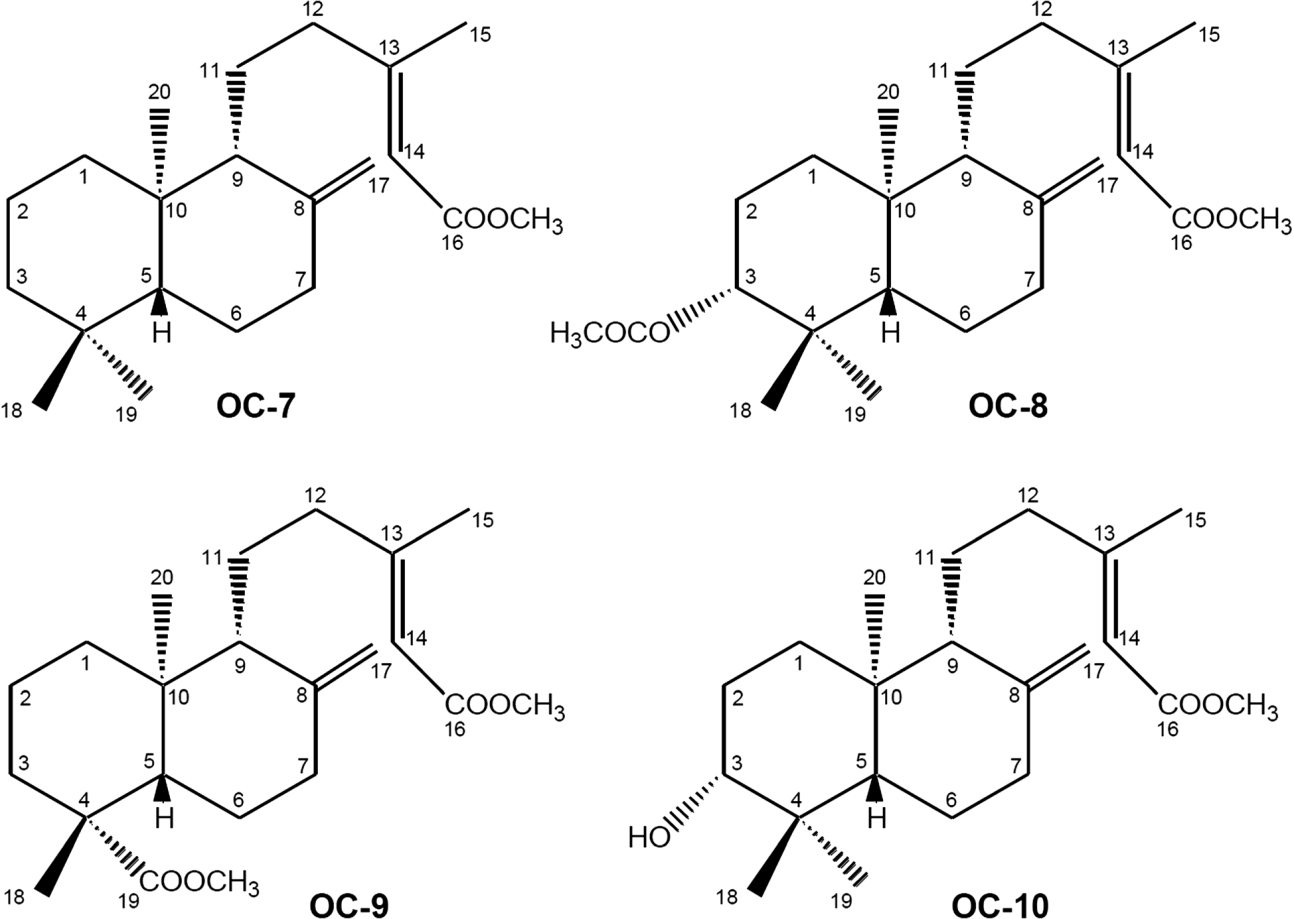
Chemical structures of the methylated compounds

In the present study, we have determined the Minimum Inhibitory Concentration (MIC) of (1) the *C. lansgsdorffii* oleoresin, (2) one sesquiterpene and five diterpenes isolated from fractions of the *C. langsdorffii* oleoresin, and (3) four semisynthetic compounds. Table 1 lists the MIC values for potentially pathogenic bacteria; Table 2 summarizes the MIC values for multiresistant bacteria. Figure 1 illustrates the chemical structures of the secondary compounds obtained from *C. langsdorffii* and evaluated in this work; Fig. 2 depicts the semisynthetic compounds obtained from the diterpenes of *C. langsdorffii*.

**Table 1:** Antibacterial potential of some compounds against potentially pathogenic Gram-positive and Gram-negative bacteria

Microorganisms (ATCC)	Minimum Inhibitory Concentration - µg mL^−1^											
	Compounds											
	Oleoresin	OC-1	OC-2	OC-3	OC-4	OC-5	OC-6	OC-7	OC-8	OC-9	OC-10	PC
*K. rhizophila* (9341)	^a^	^a^	5.0	10.0	80.0	^a^	^a^	^a^	^a^	^a^	^a^	0.1
*S. pyogenes* (19615)	^a^	^a^	3.0	5.0	60.0	^a^	^a^	^a^	^a^	^a^	^a^	0.1
*S. pneumonie* (6305)	^a^	^a^	2.0	5.0	50.0	^a^	^a^	^a^	^a^	^a^	^a^	0.2
*E. hirae* (10541)	^a^	^a^	15.0	100.0	^a^	^a^	^a^	^a^	^a^	^a^	^a^	0.4
*S. aureus* (9144)	^a^	^a^	12.0	20.0	180.0	^a^	^a^	^aa^	^a^	^a^	^a^	0.4
*S. aureus* (6538)	^a^	^a^	15.0	18.0	^a^	^a^	^a^	^a^	^a^	^a^	^a^	0.5
*B. subtillis* (6051)	^a^	^a^	5.0	12.0	90.0	^a^	^a^	^a^	^a^	^a^	^a^	0.1
*B. cereus* (14579)	^a^	^a^	8.0	12.0	100.0	^a^	^a^	^a^	^a^	^a^	^a^	0.1
*S. dysgalactiae* (9926)	^a^	^a^	1.0	8.0	80.0	^a^	^a^	^a^	^a^	^a^	^a^	0.4
*S. agalactiae* (27591)	^a^	^a^	2.0	10.0	90.0	^a^	^a^	^a^	^a^	^a^	^a^	0.7
*S. epidermidis* (12228)	^a^	^a^	0.5	8.0	30.0	^a^	^a^	^a^	^a^	^a^	^a^	0.5
*E. aerogenes* (13048)	^a^	^a^	^a^	^a^	^a^	^a^	^a^	^a^	^a^	^a^	^a^	0.7
*P. aeruginosa* (27853)	^a^	^a^	^a^	^a^	^a^	^a^	^a^	^a^	^a^	^a^	^a^	5.9
*E. coli* (14958)	^a^	^a^	^a^	^a^	^a^	^a^	^a^	^a^	^a^	^a^	^a^	5.9
*P. mirabilis* (29906)	^a^	^a^	^a^	^a^	^a^	^a^	^a^	^a^	^a^	^a^	^a^	5.9
*M. morganii* (25829)	^a^	^a^	^a^	^a^	^a^	^a^	^a^	^a^	^a^	^a^	^a^	5.9
*C. freundii* (8090)	^a^	^a^	^a^	^a^	^a^	^a^	^a^	^a^	^a^	^a^	^a^	5.9
*S. flexineri* (12022)	^a^	^a^	^a^	^a^	^a^	^a^	^a^	^a^	^a^	^a^	^a^	1.5

^a^Inactive in the evaluated concentration (MIC values higher than 200 µg mL^−1^); Positive Control (PC), vancomycin and gentamicin; Negative Control (5 % DMSO solution v/v) did not affect growth of the microorganisms

**Table 2:** Antibacterial activity of *ent*-copalic acid against multiresistant Gram-positive and Gram-negative bacteria

Minimum Inhibitory Concentration - µg mL^−1^		
Multiresistant Microorganisms	Copalic acid (OC-2)	Positive Control
*S. epidermidis* (ATCC 14490)	50.0	1.5
*S. epidermidis* (clinical isolate)	15.6	1.5
*S. capitis* (ATCC 27840)	50.0	0.7
*S. capitis* (clinical isolate)	31.25	0.7
*S. aureus* (WB81-USA 400)	25.0	0.7
*S. aureus* (W7749-USA 200)	25.0	0.7
*S. aureus* (ATCC 29213)	25.0	0.7
*S. aureus* (surgical wound)	15.6	1.5
*S. aureus* (catheter)	^a^	1.5
*E. faecalis* (ATCC 19433)	25.0	1.5
*E. faecalis* (clinical isolate)	15.6	5.9
*E. faecium* (ATCC 19434)	50.0	1.5
*E. faecium* (hospital outbreak)	50.0	5.9
*S. haemolyticus* (ATCC 29970)	100.0	1.5
*S. haemolyticus* (clinical isolate)	15.6	1.5
*S. pneumoniae* (clinical isolate)	31.25	2.9
*P. aeruginosa* (clinical isolate)	^a^	5.9
*A. calcoaceticus* (clinical isolate)	^a^	0.4
*E. coli* (clinical isolate)	^a^	5.9
*K. pneumoniae* (clinical isolate)	^a^	2.9

^a^Inactive in the evaluated concentration (MIC values higher than 200 µg mL^-1^); Positive Control (PC), vancomycin and gentamicin; Negative Control (5 % DMSO solution v/v) did not affect growth of the microorganisms

MIC values against Gram-positive and Gram-negative potentially pathogenic microorganisms ranged from 0.5 to over 200.00 μg mL^−1^ for the evaluated compounds. After preliminary evaluation of the MIC values (Table 1), we determined the antibacterial activity of the most promising compound—the diterpene OC-2—against Gram-positive and Gram-negative multiresistant bacteria.

According to Table 2, compound OC-2 was the most promising agent against multiresistant bacteria, with MIC values ranging from 15.60 to 31.25 μg mL^−1^ for a large number of tested microorganisms. Because exposure of *Staphylococcus aureus* (isolated from surgical wound), *Staphylococcus epidermidis* (clinical isolate), *Staphylococcus haemolyticus* (clinical isolate), *Staphylococcus capitis* (clinical isolate), *Enterococcus faecalis* (clinical isolate) and *Streptococcus pneumoniae* (clinical isolate) to OC-2 yielded promising results, we selected these strains to continue evaluating the antibacterial activity of this compound by the Minimum Bactericidal Concentration (MBC) assay (Table 3). The MBC results confirmed that OC-2, the main biomarker of the genus *Copaifera* [20], was active against the multiresistant strains *S. aureus* (surgical wound), *S. haemolyticus*, *S. capitisE. faecalis* and *S. pneumoniae*.

**Table 3:** Minimum Bactericidal Concentration of *ent*-copalic acid against multiresistant Gram-positive bacteria

Multiresistant microorganisms	Minimum bactericidal concentration µg mL^−1^	
	Copalic acid OC-2	Positive control
*S. aureus* (cirurgical wound)	15.6	1.5
*S. epidermidis* (clinical isolate)	31.25	1.5
*S. haemolyticus* (clinical isolate)	15.6	1.5
*S. capitis* (clinical isolate)	31.25	0.7
*E. faecalis* (clinical isolate)	15.6	5.9
*S. pneumoniae* (clinical isolate)	31.25	2.9

Positive Control (PC), vancomycin

The Gram-positive bacteria were more sensitive to the tested compounds than the Gram-negative bacteria, probably because these two groups of bacteria have different cell walls in which the compounds act at distinct locations. Indeed, besides the plasmatic membrane, Gram-positive bacteria bear a thick wall that consists predominantly of peptidoglycan; Gram-negative bacteria possess a stratified wall constituted of an external membrane, a fine stratum and an inner peptidoglycan-based plasmatic membrane. Bearing these features in mind, we suspect that antimicrobials have more difficulty penetrating Gram-negative bacteria: their external stratum composed of lipopolysaccharide determines surface properties like permeability and susceptibility to antibiotics. In other words, the external membrane of Gram-negative bacteria most likely acts as a barrier to active substances.

The chemical structures of the evaluated compounds revealed that modifications to the molecular structure markedly altered the antibacterial activity: the methylated diterpenes (OC-7 to OC-10) did not display any action; the diterpenes from which the aforementioned diterpenes originate (OC-2, OC-4, OC-5 and OC-6) did. Studies conducted by our research group [14, 15] confirmed this and highlighted which structural characteristics in diterpenes elicit promising antibacterial activity. For example, existence of one hydrogen-bond donor group (HBD; hydrophilic group) interacting with phosphorylated groups on the membrane of the bacterial cell seemed to be crucial. The presence of a second HBD reduced or abated the antibacterial activity of the compound. The present study confirmed this theory—we obtained results similar to those that Souza et al. [14] achieved when they used the same compounds against cariogenic bacteria. The Gram-negative bacteria were more resistant to OC-2, affording MIC values higher than 200 μg mL^−1^. One Gram-positive strain (*S. aureus*, isolated from catheter) was resistant to this diterpene, possibly because it was a more robust strain than the other strains assessed in this study.

**Fig. 3:**
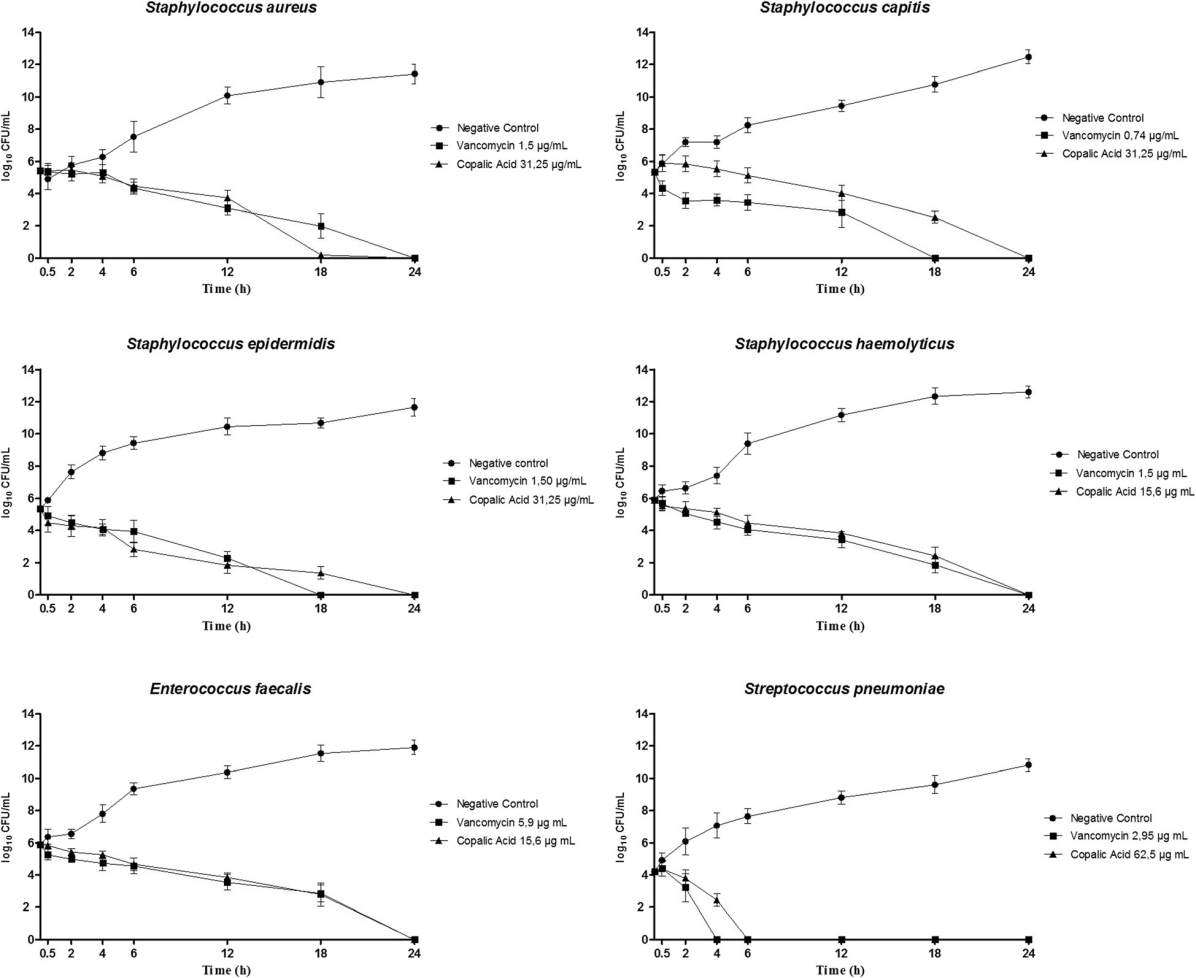
Bactericidal kinetics of copalic acid against multiresistant bacteria

We constructed time-kill curves for the selected multiresistant bacteria (Fig. 3) to verify how long it was necessary for OC-2 to completely kill the microorganisms. According to Fig. 3, OC-2 eliminated all the tested bacteria within 24 h of incubation. Inhibition of bacterial growth started to decrease after 12 h of incubation; the bactericidal potential of OC-2 became evident between 18 and 24 h.

Medoza et al. [21] stated that the time-kill curve depended on the bacterial species and on the concentration of the tested compound. The results obtained in this work agreed with the data presented by Souza et al. [13] and Souza et al. [14] when they tested copalic acid against cariogenic and anaerobic bacteria. We found that *S. pneumoniae* exhibited a distinct behavior: OC-2 eliminated this bacterium within only 6 h of incubation, which was a satisfactory result that confirmed the antibacterial activity of this compound (Fig. 1).

**Fig 4:**
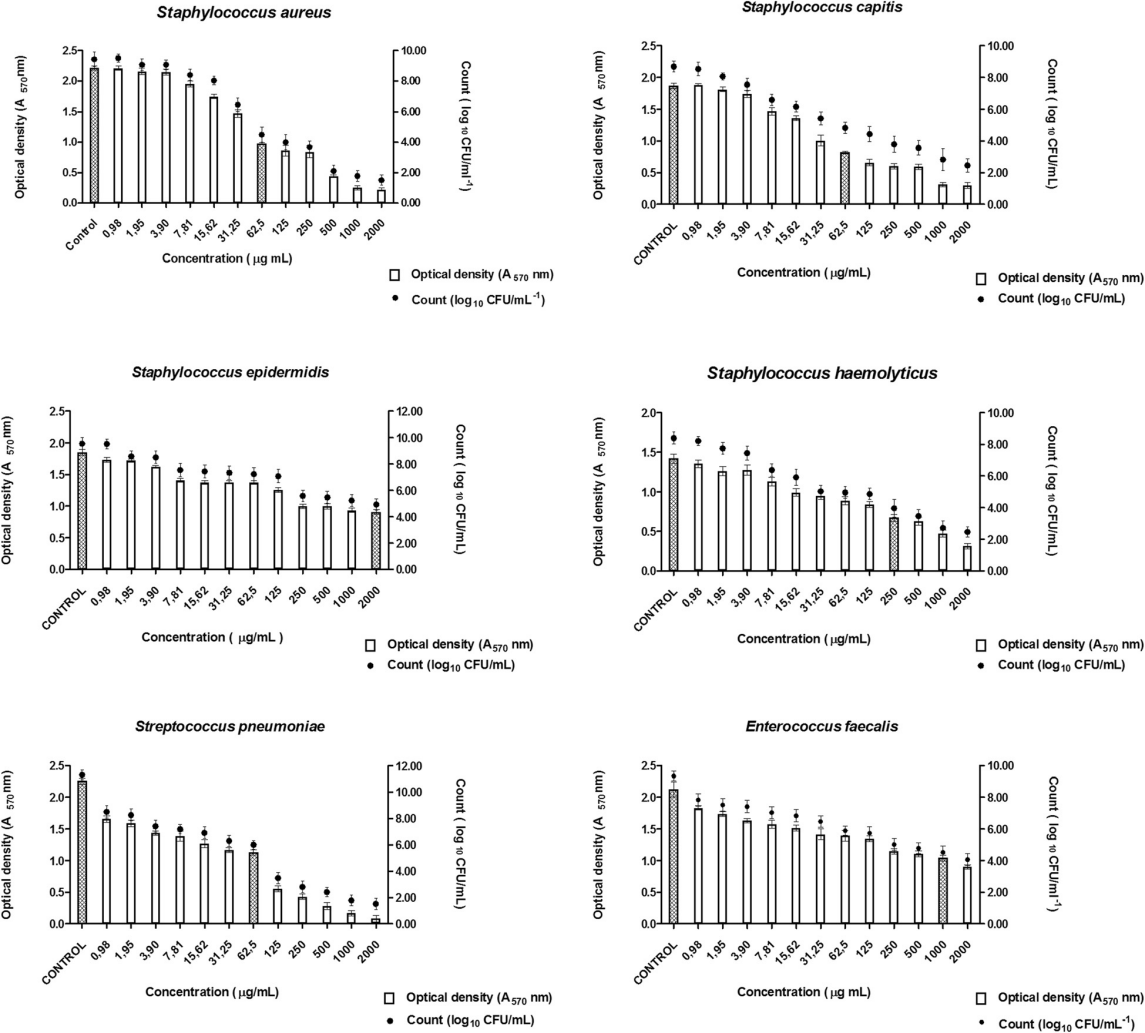
Antibiofilm activity of Copalic acid as demonstrated by optical density (A_570_) and number of microorganisms (Log_10_ CFU mL^−1^)

As for the Minimum Inhibitory Concentration of Biofilm (MICB_50_) calculated for OC-2, it ranges from 62.5 to 2000 μg mL^−1^; this compound displayed 50 % antibiofilm activity against all the tested bacteria (Fig. 4), especially *Streptococcus pneumoniae*, *Staphylococcus aureus* and *Staphylococcus capitis*. More specifically, treatment of *Enterococcus faecalis*, *Staphylococcus epidermidis*, *Streptococcus pneumoniae*, *Staphylococcus haemolyticus*, *Staphylococcus capitis* and *Staphylococcus aureus* with OC-2 furnished MICB50 values of 1000.0, 2000.0, 62.5, 250.0, 62.5 and 62.5 μg mL^−1^, respectively (Fig. 4).

To investigate the antibiofilm potential of the diterpene OC-2, we determined the MICB_50_—the smallest concentration of antibacterial agent that is able to inhibit the formation of biofilm mode by 50 % or more—by optical density and the number of microorganisms (CFU mL^−1^) (Fig. 4). Quantification consisted in using crystal violet as the coloring agent. We removed crystal violet with an appropriate solvent after a certain incubation period and measured the absorbance [18, 22]. However, this technique did not measure cell viability. Therefore, we also removed the non-adhered cells; re-suspended, homogenized and diluted the biofilm; and diluted aliquots in Brain and Heart Infusion (BHI) agar to determine the number of CFU mL^−1^. One issue was that second method could generate biofilm agglomerates, which would be difficult to dissociate in suspensions used for cell counting in agar. Despite their individual limitations, the combination of these two techniques was satisfactory: it afforded reliable results on antibiofilm activity. Literature reports have pointed out that biofilms can be 10–1000 times more resistant to antibacterial effects than a planktonic culture of the same strain [23,24]. Comparing the MIC and MICB_50_ values obtained for OC-2, this compound provided fourfold, twofold and twofold higher MICB_50_ for *Staphylococcus aureus*, *Staphylococcus capitis* and *Streptococcus pneumonia*, respectively, as compared with MIC. Ceri et al. [25] and Gursoy et al. [26] have also reported lower MIC values as compared with MICB_50_. On the basis of the present results and of the data published in Souza et al. [14], medicinal plants derivatives may pave the way for the development of new semisynthetic molecules. In this sense, OC-2 constitutes a potential agent to act against Gram-positive multiresistant bacteria that can form a biofilm.

Table 4 shows the IC_50_ values and the selectivity index obtained for the normal and the tested tumor cell lines after treatment with the *C. langsdorffii* oleoresin and OC-2. The *C. langsdorffii* oleoresin and OC-2 yielded IC_50_ values of 365.90 and 107.30 μg mL^−1^ for the normal cell line V79, respectively. The *C. langsdorffii* oleoresin was only active against the tumor cell line MCF7 (IC_50_ = 488.90 μg mL^−1^). The IC_50_ values achieved for the tumor cell lines treated with OC-2 ranged from 44.03 to 351.20 μg mL^−1^.

**Table 4:** IC_50_ values and selectivity index (IS) of a normal cell line and different tumor cell lines treated with *C. langsdorffii* oleoresin or (-)-copalic acid and the positive controls, doxorubicin (DXR), (S)-(-)-camptothecin (CPT) and etoposide (VP16)

Cell lines	*C. langsdorffii*		(-)-copalic acid		DXR		CPT		VP16	
	*IC*_*50*_ *µg mL* ^*−1*^	*IS*	*IC*_*50*_ *µg mL* ^*−1*^	*IS*	*IC*_*50*_ *µg mL* ^*−1*^	*IS*	*IC*_*50*_ *µg mL* ^*−1*^	*IS*	*IC*_*50*_ *µg mL* ^*−1*^	*IS*
V79	365.90	-	107.30	-	7.83	-	19.61	-	45.90	-
U343	ND	-	222.50	0.48	0.70	11.18	5.71	3.44	2.18	21.05
U251	ND	-	275.20	0.38	16.28	0.46	11.14	1.76	42.97	1.06
M059J	ND	-	68.31	1.57	6.98	1.12	15.55	1.26	2.18	21.05
MCF 7	488.90	0.74	150.30	0.71	5.39	1.45	36.09	0.54	82.67	0.55
HepG2	ND		351.20	0.30	62.13	0.12	11.87	1.65	235.37	0.19
HeLa	ND		44.03	2.43	21.90	0.35	19.38	1.01	225.50	0.20
B16F10	ND		257.60	0.41	3.81	2.05	20.17	0.97	48.91	0.98

Normal cell line: Chinese hamster lung fibroblasts (V79). Tumor cell lines: murine melanoma (B16F10), human breast adenocarcinoma (MCF-7), human cervical adenocarcinoma (HeLa), human hepatocellular liver carcinoma (HepG2), and human glioblastoma (MO59J, U343 and U251). ND, not determined. The selective index is the ratio between the IC_50_ values of the oleoresin or (-)-copalic acid on V79 cells and those found in the cancer cell lines

The diterpene OC-2 exerted a more pronounced antiproliferative effect on the HeLa tumor cell line, IC_50_ = 44.03 μg mL^−1^, which was significantly lower than the value obtained for the normal cell line V79 (IC_50_ = 107.30 μg mL^−1^) and upon treatment of HeLa cells with the well-known chemotherapeutic drug VP16 (IC_50_ = 225.50 μg mL^−1^). Treatment with OC-2 afforded the highest selectivity index for the HeLa cell line.

## Conclusion

Among the compounds tested in this study, the diterpene OC-2 was the most active antibacterial, especially against Gram-positive multiresistant bacteria, and it exerted the most pronounced antiproliferative effect against cancer cell lines. Therefore, this diterpene represents a potential drug to treat infections caused by multiresistant bacteria both in the planktonic and sessile mode; it is also a promising lead to develop anticancer drugs.
